# Comparison of multivariate analysis methods for extracting the paraffin component from the paraffin-embedded cancer tissue spectra for Raman imaging

**DOI:** 10.1038/srep44890

**Published:** 2017-03-22

**Authors:** Phiranuphon Meksiarun, Mika Ishigaki, Verena A.C. Huck-Pezzei, Christian W. Huck, Kanet Wongravee, Hidetoshi Sato, Yukihiro Ozaki

**Affiliations:** 1 School of Science and Technology, Kwansei Gakuin University, 2-1 Gakuen, Sanda, Hyogo, 669-1337, Japan; 2 Institute of Analytical Chemistry and Radiochemistry, CCB – Center for Chemistry and Biomedicine, Leopold-Franzens University, Innrain 80/82, 6020 Innsbruck, Austria; 3 Sensor Research Unit, Department of Chemistry, Faculty of Science, Chulalongkorn University, 254 Pathumwan, Bangkok, 10330 Thailand.

## Abstract

This study aimed to extract the paraffin component from paraffin-embedded oral cancer tissue spectra using three multivariate analysis (MVA) methods; Independent Component Analysis (ICA), Partial Least Squares (PLS) and Independent Component - Partial Least Square (IC-PLS). The estimated paraffin components were used for removing the contribution of paraffin from the tissue spectra. These three methods were compared in terms of the efficiency of paraffin removal and the ability to retain the tissue information. It was found that ICA, PLS and IC-PLS could remove the paraffin component from the spectra at almost the same level while Principal Component Analysis (PCA) was incapable. In terms of retaining cancer tissue spectral integrity, effects of PLS and IC-PLS on the non-paraffin region were significantly less than that of ICA where cancer tissue spectral areas were deteriorated. The paraffin-removed spectra were used for constructing Raman images of oral cancer tissue and compared with Hematoxylin and Eosin (H&E) stained tissues for verification. This study has demonstrated the capability of Raman spectroscopy together with multivariate analysis methods as a diagnostic tool for the paraffin-embedded tissue section.

Head and neck (HN) cancers are the type of cancerous tissues located around oral cavity, pharynx and larynx^1^. The most significant factors in the development of HN cancers include alcohol consumption, tobacco use and viral infection (e.g.; HPV). As of 2012 global survey, total 300,400 of HN cancer cases were estimated and half of which resulted in deaths^2^. The total deaths toll of HN cancers from 1990 to 2013 was increased from 84,000 to 135,000 cases^3^. The most prevalence type of cancers found in oral cavity area is the oral squamous cell carcinoma (OSCC).

Chemical imaging spectroscopy is a technique where molecular and spatial information of a sample can be obtained simultaneously. The information about the tissue complex obtained can provide pathologists with tremendous helps for cancer diagnosis. Raman imaging has been reported for investigations of OSCC several times. Chen *et al*., reported the study of keratin component in OSCC tissue. By using single value decomposition (SVD) and multivariate curve resolution (MCR) analysis, it was found that the keratin component of OSCC could be used for cancer tissue discrimination^4^. Oral cancer and healthy tissues were studied by a high frequency region. The bands due to OH (3550–3350 cm^−1^) and CH (2965–2910 cm^−1^) stretching vibrations were used for tissue discrimination as the water content of cancer was found to be higher than that of tissue^5^. The nucleoli, nuclei and cytoplasm of oral cancer, pre-cancer and normal cell lines were studied using Raman spectroscopy. PCA showed that these 3 groups can be discriminated by the biochemical variation of nuclei acid, proteins and lipids^6^.

Paraffin-embedded tissue section is the standard protocol for OSCC histopathological diagnosis. The tissue dissected from a patient is usually fixed with formalin and embedded in paraffin to preserve it from the degradation. The tissue section requires a staining process for visualizing the morphology of the tissue. However, the staining process is time-consuming and the stained tissue can provide only the architecture of the cancer tissue. The problems arising from a paraffin component in the cancer tissue section were found in both FTIR and Raman study. For FTIR study of paraffin embedded melanoma tissue, paraffin component was found to be easily identified because its bands were not overlapped with the biological component bands^7^. FTIR spectral regions without paraffin signal (3360–3050, 1810–1500 and 1440–800 cm^−1^) were selected for the analysis. Rather than removing the paraffin-overlapped region, Zelgar *et al*. reported the tissue deparaffinization prior to the measurement. The paraffin removed oral squamous cell carcinoma tissue section were studied using FTIR imaging to visualize the different tissue feature. Color images generated from hierarchical cluster analysis (HCA), K-mean clustering and 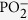 bands demonstrated the feasibility of FTIR images for tissue pattern analysis^8^,^9^. However, in the case of Raman spectra, the paraffin spectral features were found to be totally overlapped with Amide I, III and around the low wavenumber region. Mian *et al*. reported that the paraffin embedded tissues require at least 30 minutes to totally remove the paraffin^10^. However, long-term immersion of xylene could deteriorate the tissue structure. In the studies of embryonic chick cornea and prostrate gland using Raman spectroscopy, the paraffin regions were removed prior to the multivariate analysis to prevent the results from the contribution of paraffin^11^,^12^. Omitting these paraffin-overlapped regions could alter the whole result. Moreover, even after deparaffinization, we found that the paraffin component in Raman spectra was still present in our study. Hence, we decided to develop the method for removing the paraffin component in Raman spectra. To extract the cancer tissue properties, a paraffin component must be eliminated. Raman spectra of a paraffin-embedded skin tissue were investigated by Gobinet *et al*. and Vrabie *et al*.^13^,^14^. They reported the possibility of ICA to provide the estimator of the paraffin while PCA was unable to do due to the non-Gaussianity of paraffin. The paraffin components were successfully used for extracting the skin cancer spectra. The efficiency of paraffin estimation was limited by the fact that ICA is based on unsupervised method. We would like to introduce the application of supervised methods for improving the paraffin estimation efficiency.

The present study investigates the performance of ICA, PLS and IC-PLS for the paraffin estimation and develops Raman images of oral cancer tissues. We introduce PLS for extracting the paraffin estimator using its regression coefficient. IC-PLS is implemented and developed for higher performance for paraffin estimation. These methods are compared in terms of the paraffin removal capability and the effect on non-paraffin region for proteins, collagen, keratin. The ability of each method for removing paraffin is determined by observing the variation of the data in the paraffin region. In the next part, the effect of each method on the non-paraffin region is reported as the residual of sum of squares. The method with the highest paraffin removal efficiency and the lowest effect on non-paraffin part is chosen for spectral processing. The paraffin-subtracted spectra are used for constructing Raman images of cancer tissues. The Raman images and a H&E stained tissue are compared to verify the correctness of the removal of paraffin component.

## Results and Discussion

**Figure 1:**
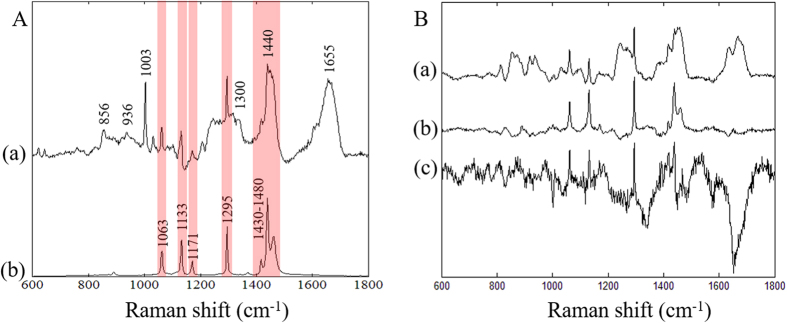
(**A**)(a), Averaged cancer tissue spectrum, (b), pure paraffin spectrum. (**B**), PCA loadings before paraffin removal are shown as PC1 (50%) (a), PC2 (9%) (b) and PC3 (5%) (c).

The cancer tissue sections from oral cancer were scanned to collect the hyperspectral data using an excitation wavelength of 532 nm to avoid the interference of a CaF_2_ glass slide. However, the fluorescence effect still remained and distorted the whole spectral shape. Baseline correction was then used to remove such interference. After that, the spectra were normalized to a phenylalanine band at 1003 cm^−1^ to observe variations in the bands due to biological components such as amide I, amide III and collagen. After the normalization, the spectra with low signal/noise were removed. The spectra thus obtained were found to have contributions from amide I (1690–1620 cm^−1^), amide III (1350–1240), keratin/collagen (960–740) and paraffin (1480–1430, 1300–1290, 1180–1125, 1069–1051 cm^−1^). Figure 1A(a) illustrates an averaged spectrum obtained from cancer tissue spectra. The pure paraffin spectrum was measured as the reference as shown in Fig. 1A(b). Note that the paraffin bands are strongly overlapped with other components throughout the whole spectra. To investigate variations in the biological components in the cancer tissue, the paraffin component must be removed.

### Paraffin removal process

**Figure 2:**
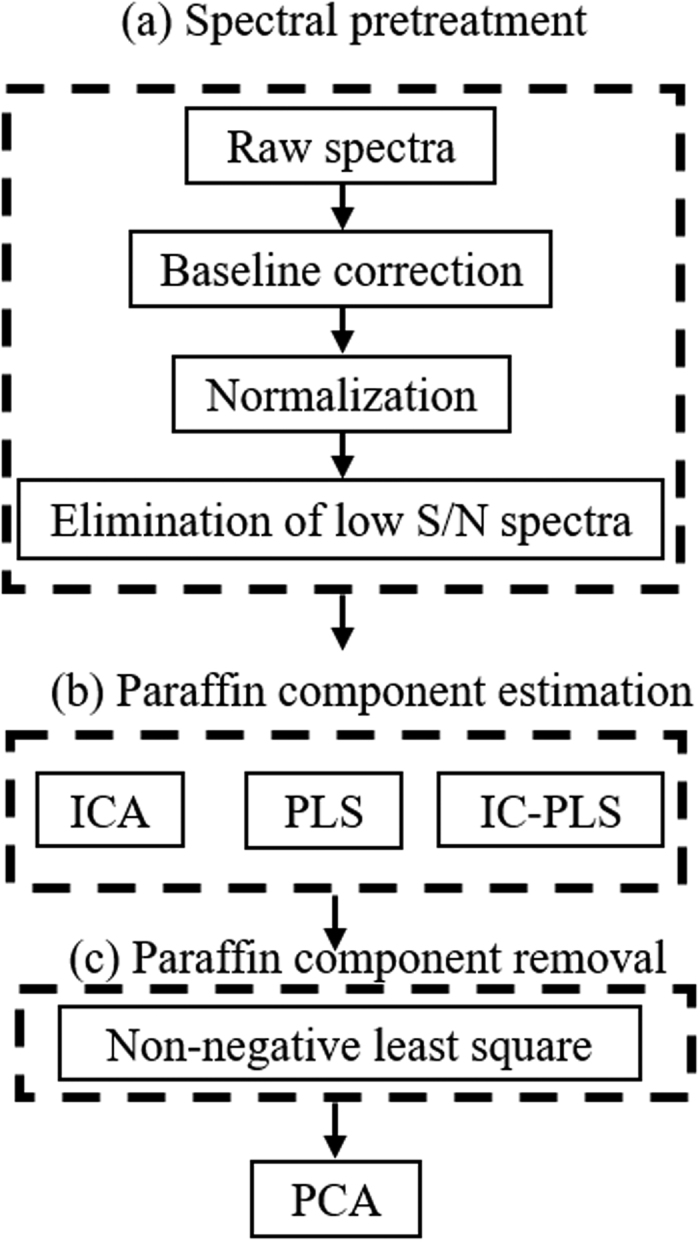
Scheme for Raman spectra analysis.

At first, a pure paraffin spectrum, shown in Fig. 1A(b), was used for removing the paraffin contribution from the cancer tissue spectra. The pure paraffin spectrum was applied to least square method for removing the paraffin bands. However, the relative intensity of paraffin in the tissue spectra was different from that in the pure paraffin spectrum as the former is distorted and unmatched to the latter. We presumed that the relative intensity of five major paraffin bands might be altered, and hence, the use of pure paraffin spectrum was unsuccessful. Then, multivariate analysis methods were employed for the paraffin component estimation as shown in Fig. 2.

We employed PCA to investigate the distribution of paraffin component in the cancer tissue. Fig. 1B depicts the PCA loading of PC 1, PC 2 and PC 3 with explained variance of 50%, 9% and 5% respectively. The paraffin bands in the region of 1480–1430, 1300–1290, 1180–1125, 1069–1051 cm^−1^ are heavily overlapped with bonds arising from other components including; Amide I (1690–1620 cm^−1^), CH_2_ bending (1480–1430 cm^−1^) and Amide III (1350–1240 cm^−1^). From Fig. 1Ba–c, paraffin bands appear in all the PCA loadings which is possibly due to the non-Gaussianity of paraffin component. The measurement of biology variable is deemed to be normally distributed. However, as the tissues used in this study were treated with several solvents and especially paraffin wax, these components were not normally deviated. Therefore, the paraffin bands could not be separated because PCA relies heavily on the Gaussian distribution of the whole data set.

**Figure 3:**
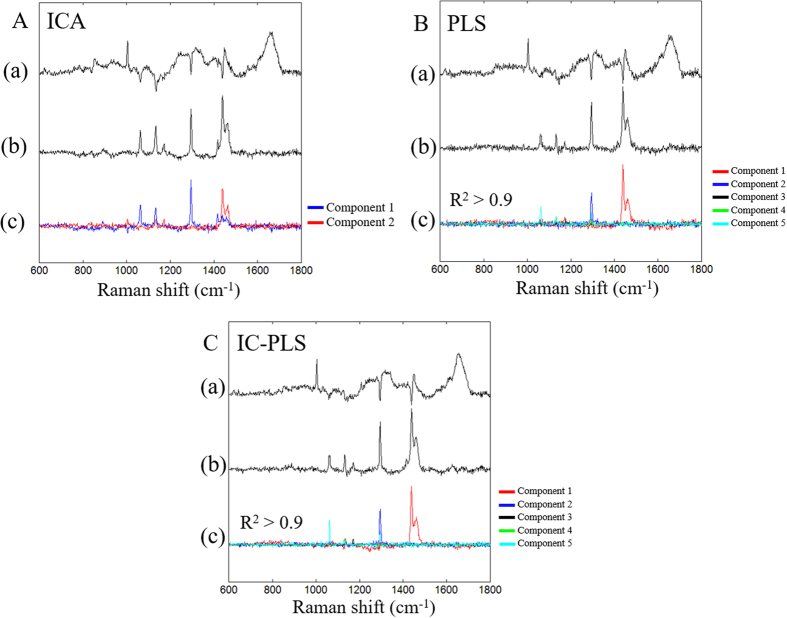
(**A**–**C**)(a), Raman spectra after paraffin removal, (**A**–**C**)(b), difference of before - after paraffin removal process and (**A**–**C**)(c), estimated paraffin components from ICA, IC-PLS and PLS.

The implementation of ICA for extracting the paraffin component from paraffin-embedded cancer tissue section was reported by Vrabie *et al*.^13^. In this study, FastICA algorithm was employed to estimate the paraffin component in the cancer tissue spectra. Before applying ICA, the whole data set was analyzed using PCA to obtain the orthogonal components. PCA loadings obtained were then used for extracting the independent components. In the first step, only one component extracted from the data was selected and used for removing the paraffin contribution from tissue spectra. The paraffin components separated by ICA shown in Fig. 3A(c) resembles that of pure paraffin. The estimated paraffin component was then used for subtraction by NNLS. After the first component was removed from the spectra, the data was again applied to ICA and NNLS for extracting and removing paraffin peaks until the paraffin peaks were disappear from any of ICA component. In this study, we found that two components were able to remove the paraffin bands. It should be noted that as the paraffin components obtained from ICA were not found in other components unlike the PCA loading where paraffin bands were found all over the loading (PC 1–3) as shown in Fig. 1B. It can be implied that the paraffin distribution in the samples is non-Gaussian. The residual from before/after paraffin removal process is shown in Fig. 3A(b).

To compare supervised/unsupervised methods, PLSR was employed for paraffin removal process following these steps. At first, the peak areas of five paraffin corresponding bands (1069–1054, 1140–1125, 1178–1162, 1298–1291 and 1470–1420 cm^−1^) were used as the dependent values. At first, the peak area of the first band (1069–1054 cm^−1^) was used for constructing the PLS model. The regression coefficient obtained was used as a paraffin component to remove the paraffin peaks from the spectra using NNLS. Paraffin-removed spectra were again used for constructing the PLS model; this process was repeated until all 5 bands were removed. We found that each regression coefficients from five paraffin peaks (R^2^ > 0.9) obtained are mostly unrelated as depicted in Fig. 3B(c). The regression coefficients shown were scaled with their concentration calculated from NNLS to show their actual intensity used for the subtraction. This result shows the interesting point that even though these five components come from the same paraffin, these peaks are relatively independent since the bands observed from each component can barely be found in other components. This result agrees with our assumption as the relative intensity of five paraffin peaks were altered.

The implementation of ICA to Non-Linear Iterative Partial Least Squares (NIPALS) algorithm so called IC-PLS was originally developed for extracting information from sensory attributes of cheese and tomato^15^. The combination of ICA and PLS model could yield a higher explained variance for non-Gaussian responses than PLS alone. By applying the ICA to loading weight of PLS, the first component of IC-PLS model was able to explain Y variance (90%) more than PLS (77%). The method was determined to build the model which yielded the highest relevance to non-Gaussian distribution of **Y** variable. IC-PLS algorithm starts with the calculation of NIPALS to determine the optimal rank, i^th^. The loading weights, 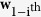, were applied to ICA to be rotated in the non-Gaussian subspace. ICA loading weights, **w**_ICA_, were then used for further calculation following Equations (5, 6, 7, 8). The overall calculation was conducted in the similar manner to PLS where five regression coefficients were obtained.

The residuals between before/after paraffin removal from ICA, PLS and IC-PLS are shown in Fig. 3A–C(b). The residual spectra and pure paraffin appear to be similar with eyes. However, some regions which are not related to the paraffin are found to be altered by each method to some extent. In the next section, efficiency for removing paraffin by these methods and their effects on non-paraffin region are discussed.

### Comparison of paraffin removal methods

**Figure 4:**
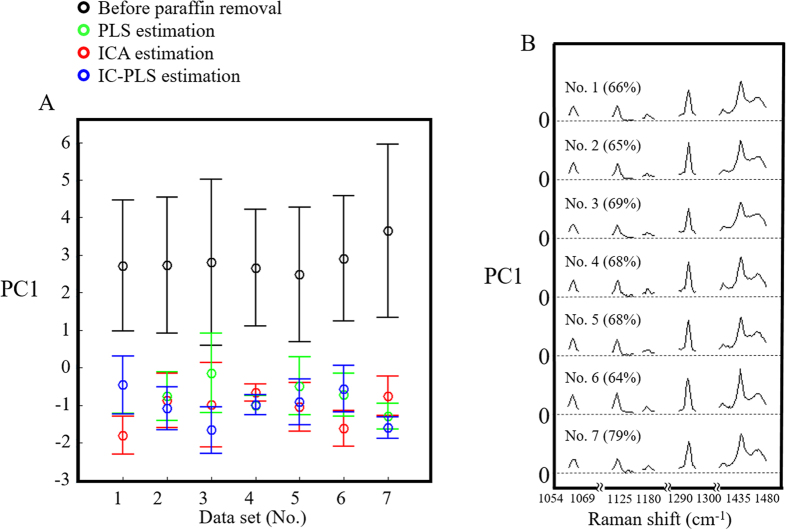
(**A**) PCA score of paraffin region from 9 data sets. Averaged and standard deviation of PC1 score of Raman spectra before paraffin removal (○), PLS (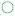), ICA (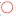) and IC-PLS (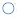) indicate the paraffin contribution. (**B**) PC1 loading shows the paraffin contribution of each data set, B.

The efficiency of paraffin removal methods was compared using PCA by comparing the paraffin influence onto the spectral variation. Raman spectra after paraffin removal process (ICA, PLS and IC-PLS) were analyzed together with the spectra before paraffin removal using the paraffin regions. The average PC1 score of all data sets are shown in Fig. 4A. The average scores of the data sets before paraffin removal are much higher than the paraffin removed data sets which are located below 0. As the paraffin removed spectra contain relatively smaller contribution from paraffin peaks, the variance due to the paraffin peaks is then shown in PC1 of PCA loading plot with explained variance of 65–79%, see Fig. 4B. The average scores of PC1 which are all in the negative region suggests the capability of the paraffin-removal methods to remove the paraffin component. However, there is no decisive result for indicating the best method for paraffin removal since all the scores are almost overlapped. We assume that all the techniques are almost equivalent in terms of the estimation of the paraffin components either using ICA, PLS or IC-PLS.

**Figure 5:**
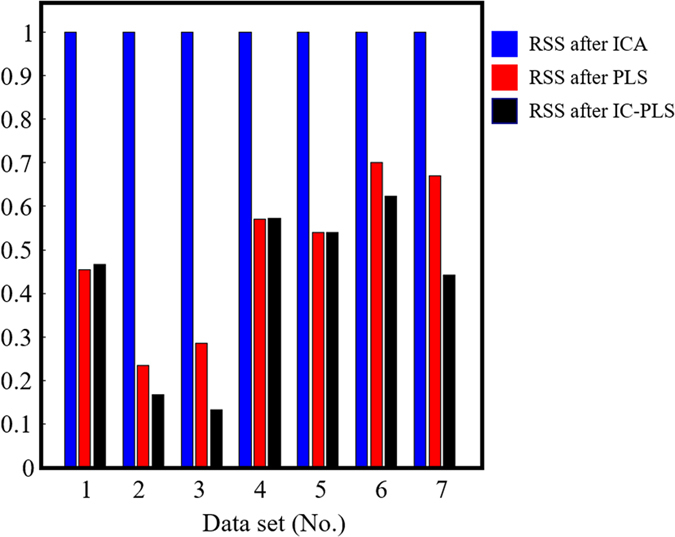
Relative residual sum of squares value of non-paraffin region of ICA (◾), PLS (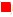) and IC-PLS (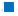).

As mentioned above that the paraffin component estimation might include the biological component region, and thus, the error in the non-paraffin bands (1670–1570, 1280–1200 and 1000–400 cm^−1^) were considered. Figure 5 illustrates relative residual sum of squares of non-paraffin region between before/after paraffin removal process. The errors of PLS and IC-PLS were normalized to that of ICA for comparison. It shows that RSS of ICA are much higher than PLS and IC-PLS. By using ICA, we found that components contain not only paraffin information but also include the other regions; Amide I, Amide III and collagen/keratin bands (Fig. 3A(c)). This is possibly due to the fact that ICA is an unsupervised method which relies heavily on the distribution of the data sets. In this study, the paraffin-embedded tissues were treated with formalin, ethanol, xylene, embedded in the paraffin block and finally washed up with octane. The spectral distribution could be altered by the sample processing which is possibly the cause of sample impurity that deteriorates the efficiency of ICA. The paraffin components from PLS and IC-PLS model shown in Fig. 3B,C(c) are bound mostly only to the paraffin peak locations and left the other regions untouched. This gives PLS based model advantages over ICA. The RSS of IC-PLS are lower than PLS in four of seven cases while the other three are almost the same level. Hence, we decided to use IC-PLS method for further analysis.

### Raman imaging

**Figure 6:**
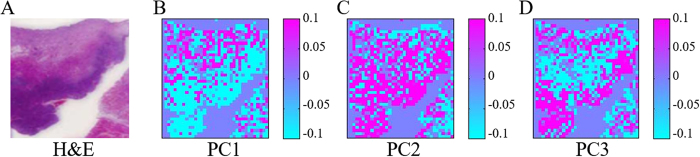
(**A**) H&E stained image and (**B**–**D**), Raman image from PC1–3 scores.

**Figure 7:**
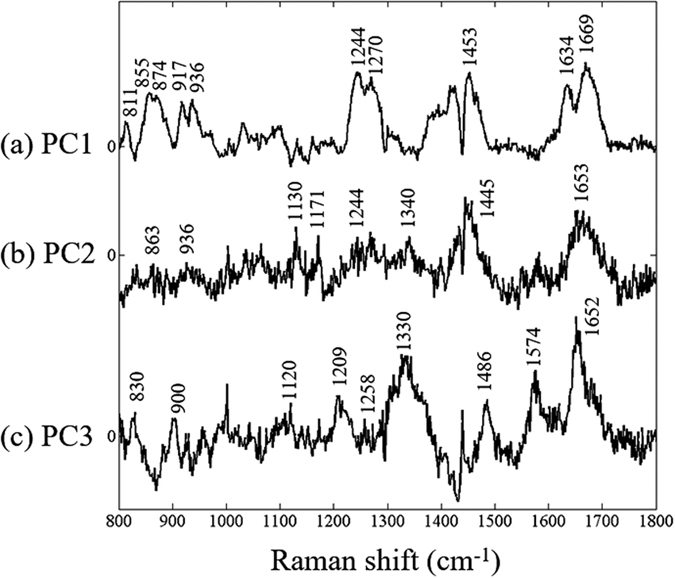
PCA loading plots for PC1–3 of oral cancer tissue section.

Raman images were constructed using the Raman spectra treated with IC-PLS. Paraffin removed spectra were analyzed using PCA. The scores of PC1–3 were used to build the pseudo color images as shown in Fig. 6B–D. The HE stained tissue image shown in Fig. 6A is used for comparison. Raman images from PC1 to 3 seem to be able to separate the cancer from the benign part where blue/pink color indicate low/high score value. It can be seen from PCA loading shown in Fig. 7 that the paraffin bands are totally removed in comparison to Fig. 1B. The band which results from the subtraction is shown around 1440 cm^−1^. This band could not affect the result much as it is prone to 0 which indicates that the low variation of this band in the spectra would not disturb the result. The explained variance of PC1 to 4 are 38%, 12% and 4% which totally explains around 54% of all the data set. The paraffin corresponding peaks did not appear in PC loading suggesting that our developed method was successfully able to remove the variation of paraffin components from the oral cancer tissue spectra.

From Fig. 7(a), PC1 loading shows the major contribution of collagen spectral profile: Amide I (1669 cm^−1^), CH_2_ bending (1453 cm^−1^), proline (1378–1234 cm^−1^), C-C stretching (936 cm^−1^), hydroxyproline ring (874 cm^−1^), proline ring (917, 855 cm^−1^) and C–O–C stretching (811 cm^−1^)^16^. We found that the PC1 score image shows consistent pattern with the HE stained tissue where the density of cancer cells is higher in the bottom part of the tissue as shown in Fig. 6B. The light blue color of scores indicates the lower amount of collagen in the cancer part than the top part of tissue which is rendered in pink. We presumed that the concentration of collagen in the oral tissue may be decreased as OSCC proliferated. The alteration of collagen contribution in OSCC was found changing from mature to immature form as OSCC progressed^17^. It was also suggested that the abnormality of collagen production in later stage of OSCC may induce the neoplastic growth^18^. The respiratory neoplasm studies reviewed that total volume of collagen was decreased as malignancy of OSCC increased^19^.

PC2 loading in Fig. 7(b) represents the alteration of protein contribution; Amide I (1653 cm^−1^), CH_2_ deformation (1445 cm^−1^), nucleic mode (1340 cm^−1^), asymmetric phosphate (1244 cm^−1^), acyl chain (1130 cm^−1^), 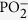 stretching (1064 cm^−1^) and C-C stretching (940–928 cm^−1^)^20^. PC2 scores image shows distinctive difference between the normal tissue (uppermost part rendered in light blue) and the cancer (pink area) which indicates the higher amount of nucleic acid/phosphate/protein. Consistent with our previous study using FTIR, the distribution of 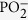 symmetric stretching of nucleic acid was found mainly in the malignant area^17^. In the study of OSCC using Raman spectroscopy, DNA, nucleic acid and protein associated components were suggested as markers for OSCC discrimination as they were found abundant in malignant tissues^21^. PC3 loading in Fig. 7(c) shows the variation of DNA component which contains Amide I (1652 cm^−1^), ring breathing modes in the DNA G, A (1574, 1486 cm^−1^), nucleic acid, phosphate and DNA related component (1330 cm^−1^), ring breathing modes in the DNA/RNA A, T (1258, 1209 cm^−1^), C-O band of ribose (1120 cm^−1^) and O-P-O stretching DNA/RNA (828 cm^−1^). These bands representing DNA alteration mark the activity DNA replication in cancer part as demonstrated in PC3 scores image (Fig. 6D). The location of DNA component found mainly at the bottom of the tissue as depicted in HE stained tissue as dark purple. Differential proteins expression was found in the transformation from oral premalignant lesions to OSCC after repeat insults using yB(a)P/DM^22^. This expression is the up-down regulation of proteins in the cells which is related to DNA replication, cell proliferation and cell cycle.

## Conclusion

In the present study, we investigated the multivariate analysis methods for removing contribution of paraffin from cancer spectra. We found that the PCA was unable to extract the paraffin component due to its distribution. The unsupervised non-Gaussian method, ICA was introduced for estimating the component. ICA could remove the paraffin bands successfully, however, its effect upon the non-paraffin region was significantly high. The supervised methods were then employed to overcome such problems. PLS with NIPALS algorithm was used for determining the paraffin corresponding variables and the regression coefficients obtained were used as a paraffin estimator. It was found that the paraffin estimated from PLS yielded equivalent paraffin removal level while providing lower RSS than ICA. We then decided to combine both methods for calculating the estimator; IC-PLS could remove the paraffin signal in the same level as other methods while giving similar/lower RSS to PLS. The paraffin-removed Raman spectra using IC-PLS was analyzed by PCA for constructing the Raman images of oral cancer. The paraffin peaks in the PCA loadings were found to be eliminated comparing to the PCA loading before paraffin removal. The paraffin removal process based on IC-PLS can be incorporated into the current protocol of Raman studies as the data pre-processing stage especially for treating the paraffin-embedded tissue^23^. The Raman images from PC1 and 3 were found to show similarity to HE-stained tissue. We found that the key Raman markers for discriminating malignant from normal tissues are collagen, phosphate and DNA. OSCC transformation from normal tissue could be observed using the suggested markers. The study has demonstrated the capability of Raman imaging and multivariate analysis for investigating the paraffin-embedded tissue. The result of our developed technique can be applied to wide range of Raman studies using paraffin-embedded tissue including dermatology, oncology or histopathological application.

## Materials and Methods

### Sample preparation

14 patients with age ranges from 32 to 73 years were given informed consent with approval of the Ethics Committee (EK 122/04) of Innsbruck Medical University. All patients signed an informed consent and permitted to the spectroscopic analysis after the project had been described in detail. The OSCC tissues dissected from the patients during the surgery were provided by the cancer biobank of the Department of Pathology at the Innsbruck Medical University. All specimens were fixed in formalin, embedded in paraffin and stained using hematoxylin and eosin (HE). The tissue sections of 4 μm were cut with microtome and placed on CaF_2_ slides (KORTH KRISTALLE GmbH, Altenholz, Germany) and glass slides (Menzel slides, Fisher Scientific, Vienna, Austria) for Raman imaging and HE histological study, respectively. The tissue sections on CaF_2_ were deparaffinised prior to Raman imaging study to reduce the influence from paraffin. The method for deparaffinization was reported elsewhere^8^. In brief, tissue sections were washed in octane by moderate shaking for 4 h in water bath and then dried at room temperature and measured. HE stained tissues were subjected for Tumor, Node and Metastasis (TNM) Staging evaluation according to the American Society of Clinical Oncology/College of American Pathologists guideline. Chemical solutions and HE staining kit were purchased from Sigma Aldrich (St. Louis, MO, USA). All experiments were performed in accordance with the approved guideline and regulations.

### Raman measurement

Raman measurements of cancerous tissue sections was carried out using inVia confocal Raman microscope (Renishaw Inc., UK). Tissue sections were placed on a motorized stage for acquiring Raman imaging spectra. The system was equipped with a Nikon objective lens (50×), a laser diode, and a CCD. The laser spot was refocused at each point to compensate the surface roughness prior to the measurement. The spectra were measured using 532 nm excitation wavelength with 10 mW at sampling point. Each sampling point was exposed to the laser for 20 second to reduce the fluorescence effect. The Raman spectra were acquired in 10 seconds (5 s × 2 times).

### Data preprocessing

Fluorescence background was removed from each Raman spectrum using 6^th^ polynomial baseline correction. The spectral intensity was corrected by the normalization using a phenylalanine band (1003 cm^−1^). The low signal-to-noise spectra were removed from the normalized spectra and centered to their mean. At this point, the pretreated spectra were ready for the paraffin removal process.

### Data analysis

#### Independent component analysis (ICA)

The artificial component embedded inside the tissue can be estimated by independent component analysis (ICA) due to its non-Gaussian distribution. ICA model assumes that the mixture of data set is the linear combination of non-Gaussian sources. ICA can be calculated from the Raman spectra matrix **X**(n × m) where n and m is observations and wavenumber. The **X** matrix can be transformed into linear combination of mixing matrix **A**(n × k) and independent components, **S**(k × m) where k is number of component. The objective of ICA is to determine the unmixing matrix, **W**(k × n), which is inverse of **A** to recover the independent source, 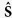
(k × m).


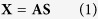



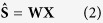


Prior to estimating independent component, the whitening process is generally performed to simplify the ICA problem. The whitened data are uncorrelated and their variance is equal to unity. PCA was used in this study to construct the linear combination of orthogonal components^24^,^25^.

#### Partial least square (PLS) regression

The algorithm for PLS used in this study is based on NIPALS. The brief explanation of NIPALS algorithm is as follows;


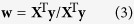


The first step is to estimate the loading weight where **X** is Raman spectra, **y** is the peak area of paraffin bands and ***w*** is normalized loading weight with length 1.


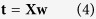



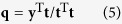



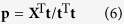


Score **t** is the linear combination of **X** and **w** which is used for calculating loading **q** and **p**.


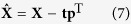



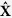
 is deflated by **p**_***i***−**1**_ and **t**_***i***−**1**_


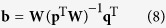


The regression coefficient **b** is used as an estimated paraffin component.

#### Independent component – partial least square (IC-PLS) regression

This method was developed by Westad as the implementation of ICA algorithm for reconstructing the loading weight of NIPALS algorithm^15^. The first step is to determine optimal rank using leave-one-out cross validation. The loading weight of 1-*i*^*th*^ component is applied to ICA for independent component calculation (Eq. 9). The extracted independent components are the rotation of NIPALS loading weight to non-Gaussian subspace.


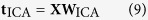


The components calculated from ICA are then used as loading weight, **W**_ICA_, for further calculation following Eqs (4, 5, 6, 7, 8).

### Non-negative least square (NNLS)

The estimated components were subtracted from the spectra using least square method with non-negative constrain.


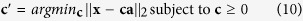


where **x**, **c** and **a** are Raman spectra, paraffin concentration profile and paraffin estimated component, respectively.**c**^**′**^ is non-negative concentration profile of **c** which yields lowest error.


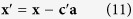


The paraffin removed spectra, **x**′, calculated by subtracting the Raman spectra with estimated paraffin component.

#### Residual sum of squares

The residual sum of squares was used to evaluate the error in the non-paraffin region.


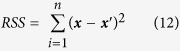


The paraffin component estimated from ICA, PLS and IC-PLS may contain not only the paraffin component but also included peaks in the non-paraffin region (1670–1570, 1280–1200 and 1000–400 cm^−1^) which deteriorate the informative part of spectra. RSS of the paraffin estimation method showing the Raman spectra in the non-paraffin region with smallest RSS was selected for further analysis in cancer imaging.

#### Raman imaging

The processed Raman spectra were used for analyzing the variations in biological components such as protein, collagen, etc. using PCA. Spectral preprocessing and data analysis were performed using Unscrambler 10.1 (CAMO Software AS., Oslo, Norway) and in-house MATLAB software (Mathworks Inc., MATLAB Version 7.1 R2010a). The FastICA package is Copyright (C) 1996–2005 by Hugo Gävert, Jarmo Hurri, Jaakko Särelä, and Aapo Hyvärinen.

## Additional Information

**How to cite this article:** Meksiarun, P. *et al*. Comparison of multivariate analysis methods for extracting the paraffin component from the paraffin-embedded cancer tissue spectra for Raman imaging. *Sci. Rep.* **7**, 44890; doi: 10.1038/srep44890 (2017).

**Publisher's note:** Springer Nature remains neutral with regard to jurisdictional claims in published maps and institutional affiliations.
